# Congenital Word Blindness, or Inability to Learn to Read

**Published:** 1914-08

**Authors:** John C. Clemesha

**Affiliations:** Buffalo, N. Y.


BUFFALO MEDICAL JOURNAL

Yearly Volume 70. AUGUST, 1914 No. 1

### ORIGINAL ARTICLES

The right is reserved to decline papers not dealing with practical medical and surgical subjects, and such as might offend or fail to interest readers. Contributors are solely responsible for opinions, methods of expression and revision of proof.

### Congenital Word Blindness, or Inability to Learn to Read.

By DR. JOHN C. CLEMESHA
Of Buffalo, N. Y.

Dr. James Hinshelwood of Glascow, first described this condition scientifically and called it congenital word blindness, some eighteen years ago. Since then articles in various journals have appeared. Among others by Kerr (1), Pringle (2), Morgan, Nettleship (3), Hinshelwood (4), Lechner (5), Thomas (6), Fisher (7), Wernicke (8), Le Comte (9), Claiborne (10), Bruner (11), Stephenson (12) and Wray (13).

Hinshelwood described word blindness as a condition, in which with normal vision, and therefore seeing letters and words distinctly, an individual is unable to interpret words or printed language. Complete or absolute word blindness is called Alexia.

Word blindness may be due to a congenital defect or deficiency in the brain centre or to some pathological process usually occurring in later life destructive to that centre.

In considering congenital word blindness we must understand that memories differ in different individuals, some being largely visual, in others auditory and the third group kinaesthetic.

The task of acquiring the visual memory of words is immensely greater than that of acquiring the visual memories of letters of which there are 52 in the English alphabet including capital and small letters. (Hinshelwood).

In these children a careful consideration and analysis of the symptoms in these cases leads directly to the conclusion that the difficulty in learning to read is due to some congenital defect in the visual memory centre for words and letters and it may be described as congenital word blindness.

In learning to read there are two distinct stages. The first stage is to store up in the visual memory centre the individual letters of the alphabet. Under normal circumstances this is done with relative ease and rapidity there being only 26 letters in our alphabet or taking capital and small letters 52 visual images to be acquired.

The memory of words is first registered in our auditory memory, i.e. we are able to spell a word before we can recognize the word by sight alone. When the individual has acquired in his visual memory a knowledge of the individual letters he is able to read words by spelling aloud each letter and thus by appealing to his auditory memory he gets the proper word, or sometimes he may simply be seen to move his lips, spelling silently each letter and thus appealing to his memory of speech movements or glosso kinaesthetic memory as called by Dr. Bastian.

To reach the second stage in the art of reading is a much more formidable task requiring for its accomplishment a much longer period of time. This second stage consists in the gradual acquirement and storage of the visual memories of words. When this is accomplished the individual reads not by analysing each word into its individual letters but by recognizing each word as a separate picture. The difficulty experienced in learning to read arises from the fact that the visual memories for words and letters are congenitally defective.

In cases of word blindness, the learning of the letters is a matter of difficulty though usually accomplished after a struggle, but the learning words is in most cases a great difficulty and yet the child can recognize the word when its kinaesthetic memories are worked as in spelling with voice and lips.

There is a definite cerebral area within which these visual memories of words and letters are registered. The angular and supra marginal gyri of the left side of the brain in right handed individuals.

There is a complete functional independence of the visual memories of letters, words and numbers as evidenced by the fact that many patients completely word and letter blind can still read figures. This is only satisfactorily explained that these memories are registered in different areas of the cortex.

In case of a person able to read several languages the letter and word visual images of each language will be grouped together forming a series of groups within the centre. If the whole centre is destroyed the person will be letterblind to all languages but if there be only a very partial interference then the word blindness may be limited to one language. The same applies to musical notes. Though word blind to a degree one may be quite apt in music.

There can also be congenital word deafness in which though the child hears words he does not understand them. Congenital word deafness is very rare but the children can be taught by lip reading how to speak.

Congenital word blindness varies in degree. Cases are not so rare as records may lead one to infer as slight cases are overlooked.

Some years ago the London County Council issued instructions to the head teachers to submit to the Medical Officer every child of the age of seven years who appeared very defective in reading and it was calculated 1 in 2000 of London Elementary school children had word blindness to a considerable degree. It was found to occur more often in boys than in girls. These children were usually bright and alert, good at figures and in fashioning articles with their hands. They would often not recognize words until spelt aloud to them and though they could recognize pictures as of a horse, fox or cow could not spell the names.

The first case reported, so far as I can find, in the English language was in the B. M. J. Vol. II, 1896, by Dr. W. P. Morgan. I can do no better than copy his report.

Percy F., a well grown lad aged 14, is the eldest son of intelligent parents. The second child in a family of seven. He has always been bright and intelligent, quick at games and in no way inferior to others of his age.

His great difficulty has been and is now his inability to learn to read. He has been at school or under tutors since he was seven years old and the greatest efforts have been made to teach him to read, but in spite of laborious and persistent training he can only with difficulty spell out words of one syllable.

The following is the result of an examination made a short time ago. He knows all his letters and can write them and read them. In writing from dictation he comes to grief over any but the simplest words. For instance I dictated the following sentence: "Now you watch me while I spin it." He wrote: "Now you word me wale I spin it," and again "Carefully winding the string round the peg" was written "Calfully winding the sturng rond the pag."

In writing his own name he made a mistake putting "Precy" for "Percy," and he did not notice the mistake until his attention was called to it more than once. I asked him to write the following words:

Song, he wrote scone.
Subject, he wrote scojock.
Without, he wrote wichout.
English, he wrote Englis.
Shilling, he wrote sening.
Seashore, he wrote seasow.

In asking him to read the sentences he had just written a short time previously he could not do so. Words such as "and" and "the" he always recognized. I then asked him to read a sentence out of an easy child's book without spelling the words. He did not read a single word correctly with the exception of "and" and "the." The other words seemed to be unknown to him and he could not even make an attempt to pronounce them.

His ability to read figures was marked. He read off quickly 785, 852, 017, 20, 969, and worked out correctly:—(a+x) (a − x) a2 − x2, and multiplied 867 by 749 correctly. He says he is fond arithmetic but that printed and written words have no meaning for him. Words written or printed seem to convey no impression to his mind and it is only after laboriously spelling them that he is able by the sound of the letters to discover their import. He had the greatest difficulty in learning his letters and no doubt originally he was letter blind, but by dint of constant application it was overcome.

Later on cases of congenital word blindness were reported where a number of children in one family were affected and in 1907 Sidney Stephenson reported a case where it had descended through three generations through the mother who was not affected.

The first case that interested me was on a boy, Stewart A., 9 years old (School 53). His vision is not good. Wears +D3 Cyl axis 90° 20/40 (figures). He cannot learn to read. Does not know F or D. T he calls R. Figures have no difficulty for him.

Then came his brother Floyd A., 13 years, School 53, Grade VI. His vision is the same as his brother and he wears the same strength of glasses. He has never been able to read and has always been sensitive about it. He is very good at figures. I tried him with the same sentences used in Dr. Morgan's case. "Now you watch me while I spin it." He wrote: "Mow you whact me while I spell it." "Carefully winding the string round the peg." He wrote: "Carefully whinning the string arround the pek." The words "song," "subject" "without," "English," "shilling," "seashore," he spelled correctly, but I caught him moving his lips when writing down those words.

The mother told me she had an older daughter, some years out of school, who could never learn to read correctly. She also told me that her mother, the grandmother of these children, never could read well.

If so this case parallels the case of Sidney Stephenson's, where he noted 6 cases in 3 generations descending through the mother who was not affected. In these cases there was imperfect vision and a certain amount of Amblyopia and Hinshelwood says 'There is no reason why a certain amount of amblyopia should not exist with word-blindness, though we can only be sure of our diagnosis when the child has healthy eyes and good visual acuity.

The next case I saw was W. H., 8 years of age, School No. 15, Attended school 3 years. While treating another member of the family the father asked me to look at the boy's eyes. He said he could not learn to read and was inclined to believe it due to laziness and inattentiveness. The boy's vision was 20/20 each eye. Figures were easy for him but he could not read easy words. Goes, does, this, that, puzzled him. Easy words as, the, cat, cow, dog, he could read. Evidently a case of congenital word-blindness to a degree.

E. E., 12 years, School 24, V Grade. Attended school 6 years. Vision, poor, with +D3 Cyl 90° 20/50. She could not learn to read. Figures were easy for her. "Now you watch me while I spin it" she wrote "Now you watch me will I spen it." "Carefully winding the string round the peg" she wrote "Careful waning the spring rond the paged." . Song she wrote correctly; subject was "subege"; without, correctly; English, correctly; shilling was "shealed"; seashore was "seasore."

Here again was a certain amount of Amblyopia, but a sum in multiplication, 867x749 she did quickly and correctly.

Clifford T., School 15, aged 9, school 3 years, is such a complete example of word blindness that I think there is also an element of tone deafness.

He is a bright and active boy. A few months ago he was examined by an expert from the east attending the meeting of the Congress of School Hygiene (Binet Tests) and declared physiologically normal for a boy of his age.

He cannot read the simplest words. He cannot spell words of three letters. The following words were given him: air, bed, cap, dig, ear, fig, gas, hot, ill, which he spelled: eir, ban, pab, dab, ear, fist,, siten, sroll, fane, respectively. He usually spells his name "Cifford" sometimes correcting it after looking at it intently.

He has a peculiar habit of commencing each word with an "s" as if altogether ignorant of the sound of the commencing syllable.

For a spelling lesson the following words were given: rode, home, horse, right, touch and wrong. He wrote: sollen, sone, sods, sond, sranet, and sonen.

(I must express my thanks for the interest his teacher, Miss Henry, took in this case and in the case of J. W. H., both boys being in her class at School 14.)

These cases are important from an educational standpoint. Taking the calculation of the London authorities that 1-2000 school children were more or less word-blind we can estimate about 50 children in the Buffalo schools who are similarly afflicted. These children are misunderstood by their teachers, their parents and others concerned in their education.

And up to the present very little application of the knowledge acquired by physiologists and psychologists of the nervous mechanism of speech functions has taken place in the practical problems of education.

### References

1. Howard Essay, 1896.
2. B. M. J., 1896.
3. Ophthalmic Review, 1901.
4. Lancet, 1900, et seq., Ophthalmic Review, 1902; Ophthalmoscope, 1904.
5. Ned Tijdschrift voor Geneeskunde.
6. Ophthalmoscope, 1905.
7. Ophthalmic Review, 1905.
8. Centralblatt fur Prakt Augenhulk, 1903.
9. Gazette des Hospitaux, 1906.
10. Journal American Association, 1906.
11. Ophthalmology, 1905.
12. Opthalmoscope, 1905-1907.
13. Wray, Lancet, 1908.

